# Matrix-Embedding Effects on Nanodiamond Phonons

**DOI:** 10.1021/acs.nanolett.5c02276

**Published:** 2025-08-20

**Authors:** Caleb Stamper, David L. Cortie, Abdulhakim Bake, Roger A. Lewis, Dehong Yu

**Affiliations:** † School of Physics and Institute for Superconducting and Electronic Materials, 8691 University of Wollongong, Wollongong, NSW 2500, Australia; ‡ Australian Nuclear Science and Technology Organisation, Lucas Heights, NSW 2234, Australia

**Keywords:** nanodiamond, nanocrystal, phonon, composite, neutron, thermal

## Abstract

A complete picture of the unique lattice dynamics of nanocrystals is slowly being painted with contributions from a variety of techniques. How these unique dynamics change when the nanocrystals are embedded in a matrix, however, is not well explored. We systematically compare the phonon spectra of diamond nanocrystals before and after embedding in a tin telluride matrix. Time-of-flight neutron spectroscopy captures phonons from elementally light nanocrystals dispersed in a heavy matrix across ∼0.5–250 meV, a challenge for other techniques. Classical molecular dynamics simulations aid interpretation. Upon embedding, surface phonons are quenched, core phonons soften, and line widths narrow due to new boundary conditions and tensile strain imposed by the matrix. Temperature-dependent measurements reveal suppressed anharmonic surface dynamics, with subtle differences between nanodiamonds in agglomerates vs isolated grains. These results are significant for understanding the vibrational and thermodynamic properties of a range of nanocrystal composites such as thermoelectric nanocomposites.

## Graphical abstract

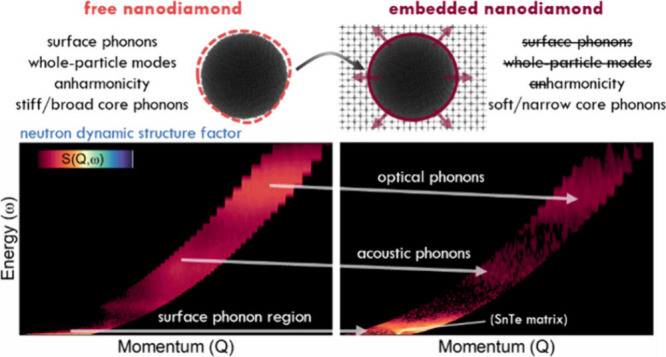

As we improve our ability to precisely manipulate material structures, we can engineer advanced materials that uniquely control the movement and interactions of wave-like particles and quasi-particles, such as mechanical vibrations (phonons).^1^ In this context, understanding the vibrational properties of nanocrystals (NCs) is of growing significance in the advancing field of phononic materials.^2^ For the effective design of NC-based phononic materials, it is important to comprehend the phononic behavior of NCs both as isolated particles and within the various chemical environments of their final bulk forms (e.g., in solution or embedded in a solid).

Many phononic materials are composed of periodically dispersed NCs in a host material or interacting NCs that form a secondary lattice.^1–3^ In these systems, the first step to comprehensively describing their vibrational properties is obtaining a precise understanding of the intrinsic vibrational properties of the individual NCs. Despite some of the unique (compared with bulk) lattice dynamics of NCs being observed two to three decades ago,^4–10^ developing a comprehensive physical picture of these dynamics has taken some time. Regardless, consistent observations of the vibrational (phonon) properties of NCs from simulation and experiment have been made, up to recent times. These include the existence of low-energy, collective modes that arise due to the mechanical freedom provided by the free particle surface, high-energy, localized vibrational modes involving individual surface atoms or ligand-like species, significant broadening of phonons in the core of the particles due to reduced lifetimes and disorder, and the softening of core phonons due to surface stresses.^11–17^ We have explored these nanoscale dynamical features in detail for ∼ 5 nm diameter diamond NCs using atomistic simulations and experiments in a forthcoming work.^18^

While there has been some recent progress in describing the lattice dynamics of individual and also ligand-joined (colloidal) NCs,^19–21^ works investigating the phonon behavior of NCs embedded in a solid matrix are extremely limited. Numerous studies include Raman measurements of embedded NCs, although most use the Raman signals merely to confirm the presence of NCs.^22^ Only a few investigations analyze the detailed phonon dynamics of embedded NCs, and these focus solely on select, high-energy, optically active phonons.^23,24^ In fact, *an experimental investigation of the effects on NC phonons upon being embedded in a solid matrix across its whole phonon spectrum has not yet been achieved* (here, we define the “whole phonon spectrum” as the energy range of mechanical vibrations on the meV/THz scale ranging from low-frequency whole-particle resonances to high-frequency optical modes). The reasons for this overlap with the challenges of studying NCs in the first place: the relative novelty of synthesizing high yields of a variety high-quality NCs, limitations on observable modes due to selection rules for optical (THz, Raman, *etc.*) methods, and the slow realization of the significance or uniqueness of phonons in nanosized systems compared to nanoscale electronic or photonic properties. Additionally, measuring NC phonons in an optically opaque solid matrix has the following challenges:

1. Optical techniques are limited in their matrix-penetration capabilities, especially for matrices with large atomic numbers.
2. Interpretation of phonon data from composite materials requires prerequisite studies of the individual host and NC lattice dynamics for the deconvolution of vibrational data, especially if there is a large (energy) overlap in phonon modes between the materials.
3. Atomistic simulations require accurate interaction potentials for the NC and host matrix and such simulations have a high cost requiring 10^3^–10^6^ or more atoms.

Despite these challenges, recent studies have made progress in measuring phonon modes of embedded NCs using various techniques: surface plasmon-enhanced Raman scattering,^25^ time-of-flight (TOF) inelastic neutron scattering (INS),^26^ and nuclear inelastic X-ray scattering (NIXS).^27^ These methods can measure the full phonon spectrum of embedded NCs. However, limitations remain: optical measurements are restricted by sample depth and atomic contrast (so far only detecting heavy NCs in relatively light SiO_2_), and no studies have yet compared embedded versus nonembedded NCs, limiting modal analysis.

In this letter, we investigate the whole phonon spectrum of elementally light nanodiamond (ND) crystals when embedded in a heavy tin telluride (SnTe) matrix, comparing them to free NDs in powder form (Figure 1). Since C has a similar neutron scattering cross section (σ_coh_^C^ = 5.55 barn) to Sn and Te (σ_coh_^Sn^ = 4.87 barn, σ_coh_^Te^ = 4.23 barn), and the ND and SnTe phonon modes are well-separated in energy, phonons from a small fraction (<1%) of NDs mixed throughout a bulk sample can be detected. Additionally, because we have thoroughly analyzed the INS signal from the ND powder sample in our forthcoming work,^18^ we can confidently evaluate the origin of differences in the powder and matrix-embedded ND signals, thus overcoming challenges 1 and 2. We also impose simple environmental conditions on ND particles in molecular dynamics (MD) without requiring additional force constants or atoms, circumventing challenge 3.

**Figure 1:**
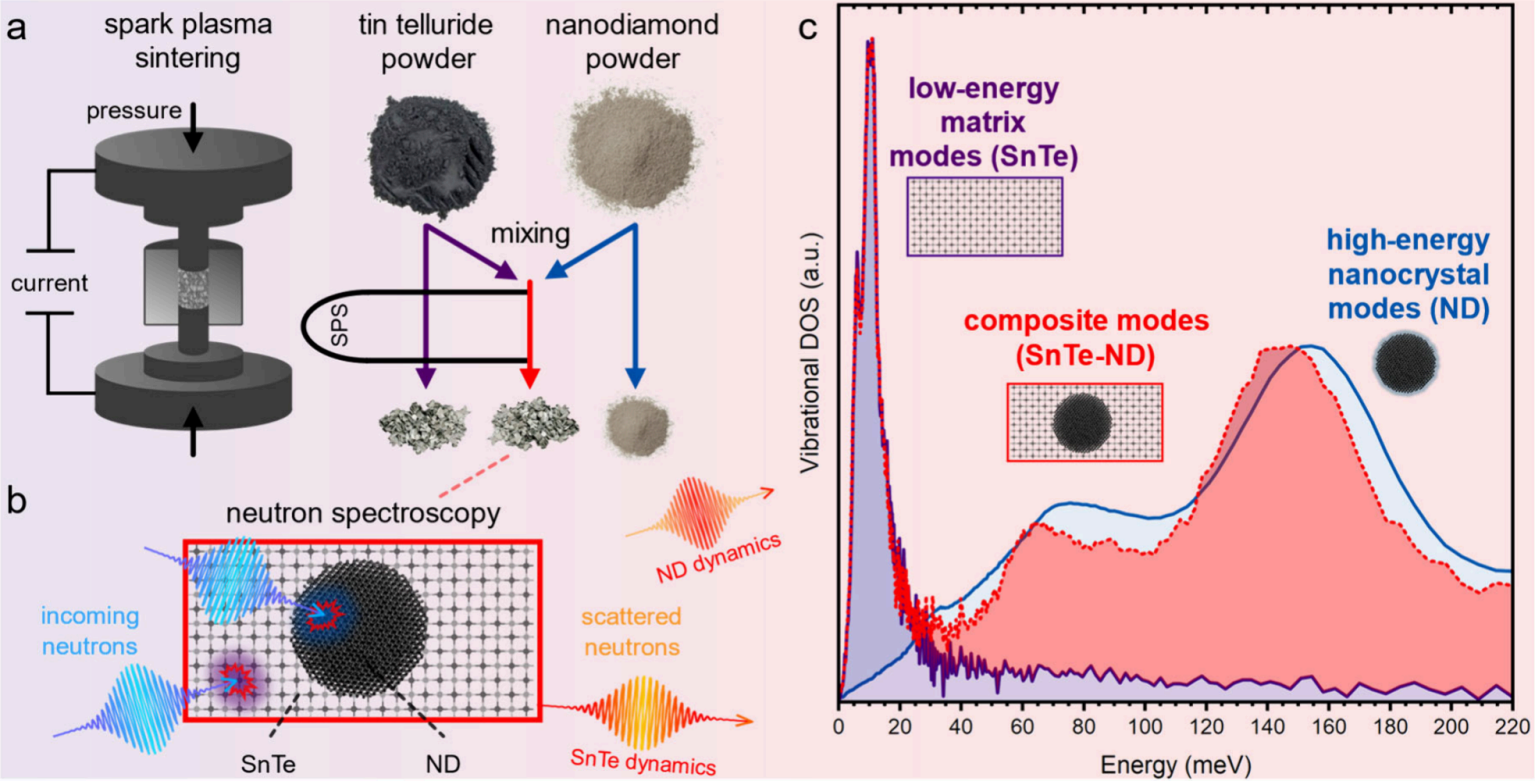
Overview of the experimental approach used to study the THz-frequency vibrational modes of nanodiamond (ND) embedded in SnTe. (a) Schematic of the three primary materials and consolidation method used in this work. (b) Schematic of neutron spectroscopy of the composite material with inelastically scattered neutrons providing dynamical information for the matrix (SnTe) and nanocrystal (ND) filler. (c) The peak-normalized vibrational density of states for the SnTe, ND powder, and SnTe-ND composite (2.4 vol % ND). Most of the SnTe and ND modes are well-separated in energy, allowing them to be easily deconvoluted.

In addition to the significance of understanding embedded ND lattice vibrations in the contexts of sensing (optomechanical properties) and thermal transport modification in metals and polymers,^28–33^ carbon nanoparticles are widely used in thermoelectric materials such as SnTe to impede thermal conductivity,^34^ and NDs are particularly effective at this.^35,36^ It is generally understood that the large mismatch in phonon modes between C nanoparticles and certain materials means that C nanoparticles can be excellent phonon scatterers, and their large surface-area-to-volume ratios result in many scattering interfaces.^37^ However, composite thermal conductivity models accounting for interface scattering still fail to accurately describe the effective thermal conductivity of many C nanocomposites, and related phonon scattering models require further development.^34^ A more nuanced picture that includes the embedded C nanoparticle dynamics is necessary to accurately describe composite thermal properties.

Figure 2 shows the dynamic structure factor maps, *S*(*Q*,*E*), obtained by INS for SnTe, ND, and a SnTe-ND composite (2.4 vol % ND). Between 300–583 K (the temperature range used in this work), both SnTe and ND possess face-centered-cubic (FCC) crystal structures with lattice constants of approximately 6.3 Å and 3.6 Å, respectively (diffraction patterns shown in Figure S1). At low energy, all SnTe phonon modes are visible across multiple Brillouin zones (200→420 Bragg peaks) (Figure 2a). The ND powder has three distinct features: a large incoherent elastic signal, steep acoustic phonons originating from the (111) and (220) (not visible at elastic line) Bragg peaks, and diffuse, excess low-energy signal originating from surface phonons (Figure 2b).^18^ In the composite sample, the ND (111) Bragg peak and elastic incoherent signal are clearly visible, but the surface phonon signal disappears and the acoustic modes are buried beneath the SnTe modes (Figure 2c). At high energies (>20 meV), where SnTe has no occupied phonon modes (Figure 2d), the broad core acoustic and optical modes of ND (Figure 2e) are visible in the composite (Figure 2f). These data sets highlight the unique advantages of neutron TOF spectroscopy to observe phonons from a small fraction of light NDs embedded throughout the bulk of a heavy matrix across a wide energy range which is not possible, for example, with optical techniques.

**Figure 2:**
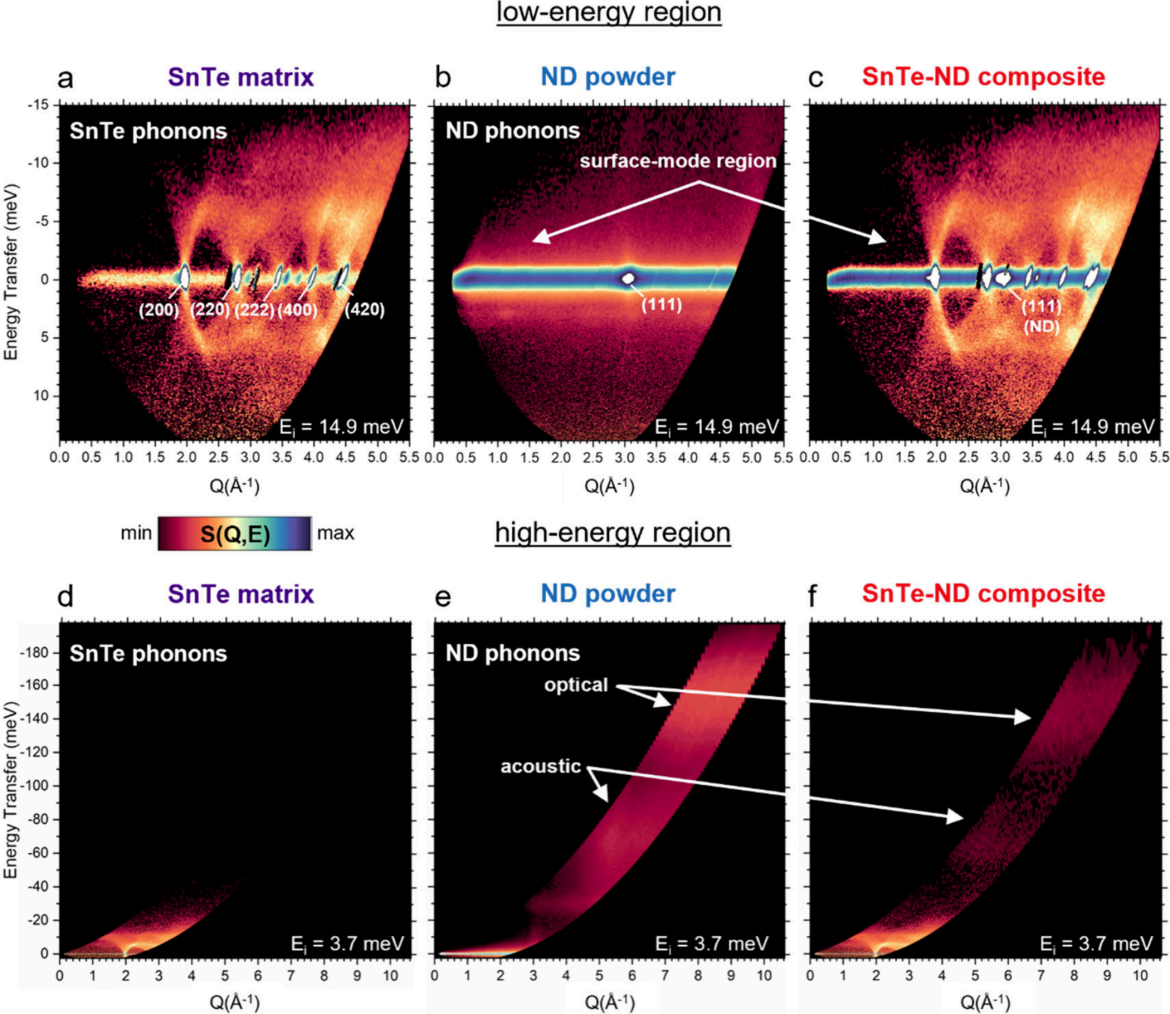
Phonon signal from a small amount of elementally light nanodiamond (ND) particles can be clearly observed in an elementally heavy, bulk, semiconductor matrix (SnTe) using time-of-flight neutron spectroscopy. The top row (a–c) shows *S*(*Q*,*E*) maps (log-scale) for the three samples corresponding to the VDOS in Figure 1b at low energies using a neutron incident energy of *E*_*i*_ = 14.9 meV. The bottom row (d–f) shows *S*(*Q*,*E*) maps at higher energies using *E*_*i*_ = 3.7 meV. All measurements were taken at 583 K. A comparison of *S*(*Q*,*E*) between ND and bulk diamond is shown in Figure S2 to further clarify the ND features.

To separate the differences in lattice dynamics between the ND powder and embedded NDs, the matrix (SnTe) VDOS is subtracted from the composite (SnTe-ND) VDOS to obtain the embedded ND VDOS. The background-subtracted embedded ND VDOS is shown in Figure 3a, along with the ND powder VDOS and bulk (microdiamond) reference, highlighting the nanoscale features of the ND powder. The Van Hove singularity (VHS) from the optical modes is fitted with a Gaussian function for a general quantitative analysis of the change in energy and broadening of modes (data in Table 1). Numerous studies have shown that a variety of NCs exhibit an excess of vibrational modes at low energy that scales linearly with energy and originates from the surface of the particles.^5,8,9,12,14–17^ In our forthcoming work, we show that the excess, linearly scaling, low-energy VDOS originates from the existence of Rayleigh surface phonons (surface acoustic waves) in ND.^18^ The general NC VDOS can be separated into two components. The first is the VDOS from the atoms at the core of the NC. This closely resembles the (low-energy range) VDOS of a 3D-Debye system with phonon dispersion ω = *v*_*s*_ | ***q*** |:

1 $$\begin{array}{c} g_{\mathrm{NC}\text{−}\mathrm{core}}\left(\omega \right)\approx g_{3D\text{−}\mathrm{Debye}}\left(\omega \right)=\frac{3V\omega^{2}}{2\pi v_{s}^{3}} \end{array}$$

where *v*_*s*_ is the speed of sound and ***q*** is the wavevector. The second is the VDOS from the atoms on the surface of the NC. This closely resembles the (low-energy range) VDOS of a 2D-Debye system at low energy with phonon dispersion ω = *v*_*R*_ | ***q*** |:

2 $$\begin{array}{c} g_{\mathrm{NC}\text{−}\mathrm{surface}}\left(\omega \right)\approx g_{2D\text{−}\mathrm{Debye}}\left(\omega \right)=\frac{A\omega }{2\pi v_{R}^{2}} \end{array}$$

where *v*_*R*_ is the Rayleigh phonon speed (*V* and *A* are the crystal volume and area, respectively). For ∼ 5 nm NDs, the core phonon modes persist close to the edge of the particles, with decreasing coherence at the edge, while the Rayleigh modes decay exponentially from the surface such that they have vanishing intensity at 1–1.5 nm from the surface.^18^ We have also observed that the ND optical modes are generally slightly stiffer than the microdiamond modes due to relatively weak, but non-negligible interparticle interactions.

**Figure 3:**
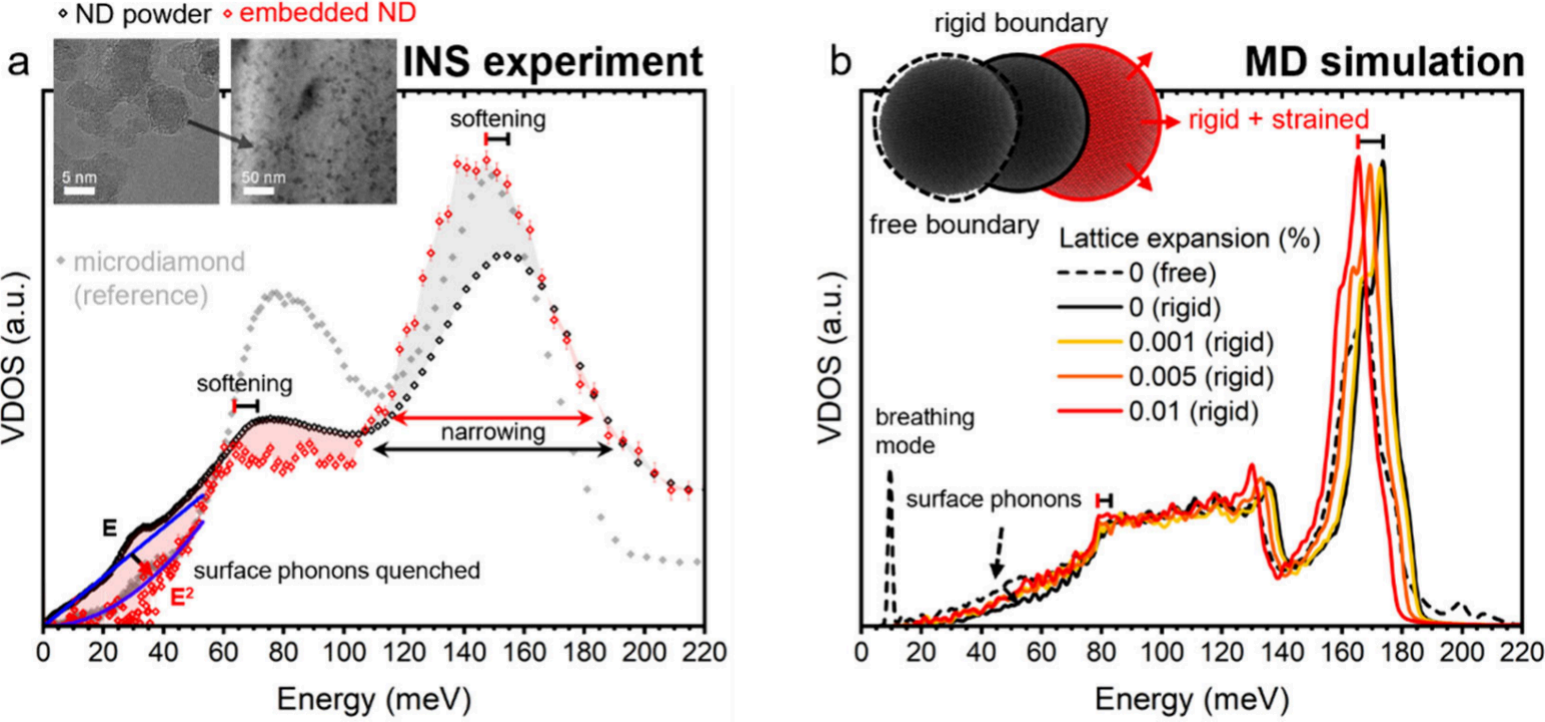
Core and surface nanodiamond (ND) phonons are altered upon compositing due to new boundary conditions and strain. (a) Inelastic neutron scattering (INS) derived vibrational density of states (VDOS) of an ND powder, ND embedded in SnTe (SnTe-subtracted), and a microdiamond (∼1 μm diameter diamond powder) reference. Transmission electron micrographs of the ND powder (left) and embedded NDs in SnTe (right) are provided as insets. (b) Molecular dynamics (MD) VDOS of a free (dotted black line), isolated ND particle, the same particle under Dirichlet boundary conditions (solid black line), and the Dirichlet particle under different degrees of tensile strain (yellow-orange lines). Parameters from Gaussian fits of the optical Van Hove singularity in (a) are presented in Table 1.

**Table 1:** Fitting Parameters of the Diamond Optical Mode Van Hove Singularity in the Vibrational Density of States of Samples Measured by Inelastic Neutron Scattering

temperature (K)	300		583		583–300 temp. difference		583 embedding difference (powder – composite)	
parameter (meV)	peak position	fwhm	peak position	fwhm	peak shift	peak broadening	peak shift	peak broadening
bulk reference^a^	-	-	147.4 (3)	49 (1)	-	-	-	-
ND powder	159.8 (3)	76 (1)	153.2 (2)	73 (1)	–6.6 (5)	–3 (2)	-	-
ND-SnTe 2.4%	145 (1)	60 (2)	146.8 (5)	60 (1)	2 (2)	0 (3)	–6.4 (7)	–13 (2)
ND-SnTe 1.0%	-	-	150.1 (2)	64 (1)	-	-	–3.1 (4)	–9 (2)

a Microdiamond powder.

Upon embedding the NDs in SnTe through spark plasma sintering (SPS), we observe three key changes in the VDOS: (1) The low-energy surface modes disappear, and the VDOS more closely resembles the microdiamond sample with its g_3D‑Debye_ (ω) ∝ ω^2^ scaling, (2) the entire VDOS softens, and (3) the full width at half-maximum (fwhm) of the Gaussian peak narrows (i.e., there is a reduction in the broadness of features) to a value in between that of the microdiamond and ND powder.

To pinpoint the origins of these changes, molecular dynamics (MD) simulations were run on a single spherical ND particle—as per ref 18—and the influences of rigid boundary conditions and strain on the resulting VDOS tested (Figure 3b). The isolated particle (dotted line) exhibits distinct features associated with the free motion of the particle surface including a low-energy resonant (Lamb) breathing mode (not prominent in INS data) and excess VDOS from surface Rayleigh phonons. When Dirichlet boundary conditions are imposed to approximate strong bonding with a heavy lattice (black line), these features are quenched and the fwhm of the optical VHS is reduced, resulting in a VDOS more reminiscent of bulk diamond. Both changes correspond well with the changes observed in the INS ND VDOS. Additionally, the phonon modes of the rigid simulated ND are stiffened due to the residual positive (compressive) internal pressure (Figure S3) caused by the new boundary conditions. The reduction in the fwhm suggests that the optical mode broadening in the free ND partly originates from the free ND surface. This is likely because the free surface allows for the occupation of modes with a wider range of energy and increased anharmonicity (discussed further below). Previous work has demonstrated NC mode-broadening arising from particle–particle interactions.^38^ Although this may partially contribute to broadening in the real powder, particle–particle interactions are ruled out as the primary broadening mechanism here as they are not present in the MD simulations. The residual excess fwhm of the embedded/rigid ND over the microdiamond sample is likely caused primarily by reduced phonon lifetimes in the NDs due to boundary scattering.^6^ To approximate the strain observed from the shift in the embedded ND Bragg peak (Figure 4c), uniform tensile (negative) stress is applied to the rigid-boundary particles (yellow to red lines). This strain arises from the lattice constant mismatch between the SnTe and ND and leads to significant mode softening. These two simple conditions—rigid boundary conditions and uniform tensile stress—mimic the environmental changes and qualitatively reproduce the differences observed in the INS data between free NDs (powder) and NDs embedded in SnTe remarkably well.

**Figure 4:**
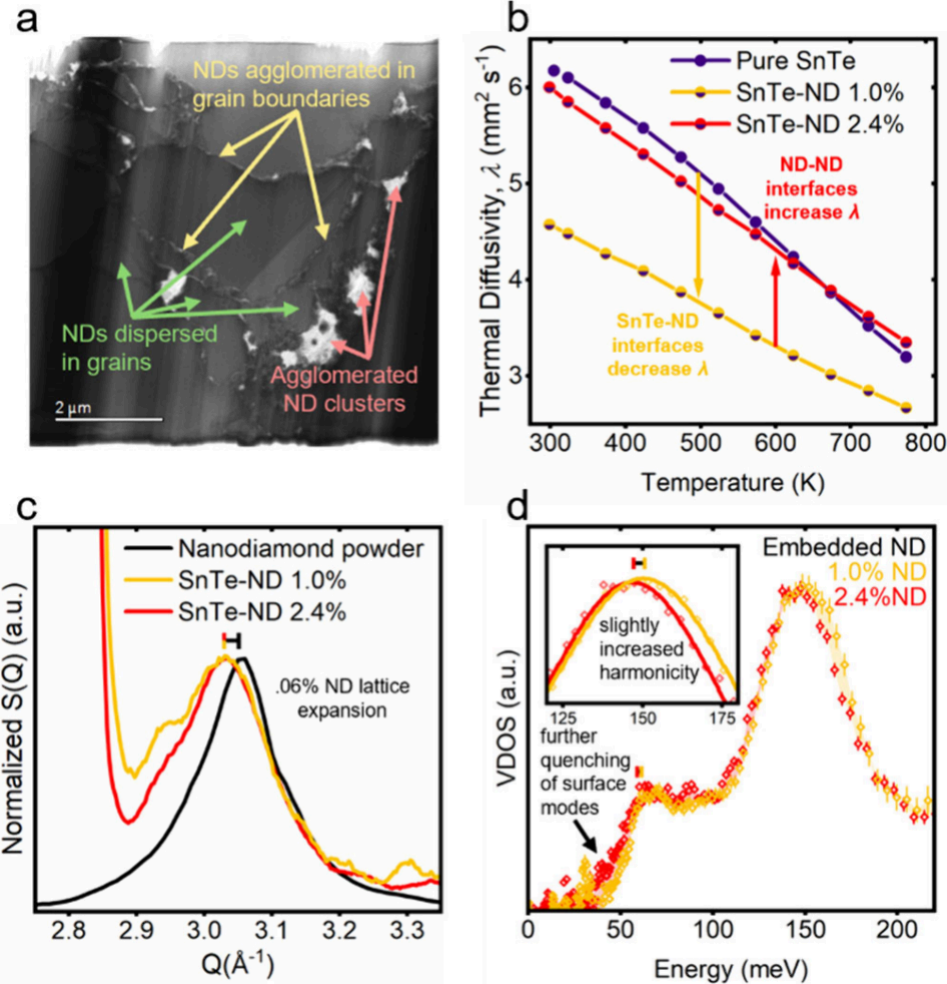
Differences in embedded nanodiamond (ND) dynamics in different local environments. (a) Scanning transmission electron micrograph (STEM) of a lamella from the SnTe-ND 2.4% sample showing various ND environments. (b) Thermal diffusivity data of the samples taken by light flash analysis highlights the larger ratio of NDs embedded in the SnTe grain in a lower concentration (1.0 vol %) SnTe-ND composite. (c) Neutron diffraction data showing the shifting of the ND (111) Bragg peak for embedded NDs compared to the ND powder. (d) Slight changes in the average ND dynamics are observed in the neutron-derived vibrational density of states (VDOS) in the 1.0% sample due to the reduced degree of ND agglomeration.

From transmission electron microscopy (TEM) data, we can see that the NDs take up a range of different local environments in the SnTe-ND sample presented thus far (2.4 vol % ND) (Figure 4a). There are three primary ND environments: embedded within the SnTe grain, within large ND channels in SnTe grain boundaries, or within large ND agglomerates/clusters. The way that NDs are dispersed throughout the matrix is expected to have a significant impact on thermal transport properties since SnTe-ND interfaces are highly efficient phonon scatterers,^34^ while ND-ND interfaces should provide efficient thermal conduction pathways due to the large intrinsic thermal diffusivity of diamond. Figure 4b shows that at 1.0% ND concentration in SnTe, thermal diffusivity is significantly reduced compared to pure SnTe due to the large number of SnTe-ND interfaces. However, at 2.4%, diffusivity significantly increases from 1.0% due to the large number of ND-ND interfaces. Despite the greater proportion of NDs dispersed in the SnTe grains in the 1.0% sample compared to the 2.4% sample which has a large degree of agglomeration, the location of the (111) diamond Bragg peak is shifted to the same degree in both samples (Figure 4c), indicating similar degrees of average ND stress. This lattice expansion is equivalent to ∼ 800 MPa of tensile hydrostatic pressure (determined using the Vinet equation as presented in Figure 2 of ref 39). In the different local ND environments (dispersed-in-grain or agglomerated), it is anticipated that the NDs exhibit different vibrational dynamics. In each INS experiment, vibrations are sampled for NDs in all their chemical settings, and deconvoluting the signal from each is not feasible without additional measurements. Nonetheless, by measuring samples with different ND inclusion homogeneities, we can observe differences in the *average* local ND environments and determine whether the different environments lead to different dynamics. Despite the variations in ND dispersion between the samples, the embedded ND VDOS curves in Figure 4d exhibit only subtle differences. The 1.0% sample shows a slight further reduction in low-energy modes associated with ND surface vibrations compared to the 2.4% sample. We hypothesize that NDs in the agglomerates have some degree of surface freedom, resulting in a small enhancement in the low-energy VDOS from surface phonons that is reduced when the ratio of dispersed NDs to agglomerated NDs increases. Comparing the Gaussian-fit optical VHSs, the 1.0% sample has a similar fwhm value, but the optical modes stiffen slightly compared to the 2.4% sample (Table 1). The stiffening of the 1.0% sample phonons is unexpected from a strain perspective since it would imply that the NDs in this sample are subject to some compressive force. If anything, an increased average tensile force might be expected due to the improved dispersion of NDs in the 1.0% sample. Regardless, the coinciding (111) Bragg peak positions of the two samples confirm that the 1.0% sample phonons are not stiffened by strain. In the following paragraph, we show that the phonons of free NDs are anharmonic compared to embedded ND phonons due to their surface freedom. Therefore, it is possible that the agglomerated NDs in the 2.4% sample exhibit slight softening due to their relative anharmonicity compared to the well-dispersed NDs in the 1.0% sample that are constrained in SnTe.

The vibrational properties of NDs are important over a wide range of temperatures, and observing the behavior of modes at different temperatures can provide insights into bond (an)­harmonicity. We took INS measurements at 300 and 583 K over which the thermal conductivity of bulk diamond roughly halves (from ∼ 1,800 to ∼ 1,000 W m^–1^ K^–1^) due to increased phonon–phonon scattering.^40^ As shown in Figure 5a, the ND powder VDOS softens significantly at the higher temperature (∼7 meV optical mode shift), and this behavior is well replicated by MD simulations, albeit the shift is slightly less (∼3 meV shift of the same modes), as shown in Figure 5b. In contrast, Figure 5c shows that when the NDs are embedded in SnTe (2.4% ND sample), this softening behavior is quenched, and the observable shift is zero, within error. Figure 5d shows the MD replicating this result; when boundary conditions are imposed on the isolated ND, the mode softening is restricted (<1 meV). *Ab initio* MD simulations suggest that bulk diamond experiences similarly small (<1 meV) lattice softening over this temperature range (Figure S4) due to the highly harmonic C–C diamond bonds. This indicates that the large ND powder softening arises from the free ND surface—an intuitive result when considering the anharmonic potential energy landscape for atoms at the surface of a free particle, where many atoms are undercoordinated. Embedding the ND in a solid matrix removes this surface anharmonicity by forcing the surface atoms into close proximity of other C or Sn/Te atoms, restricting their movement, and resulting in relatively harmonic behavior. The role of surface anharmonicity of NCs has not previously been investigated in relation to thermal conductivity. This could be significant for NC composites with soft matrix-NC bonds, such as liquids or polymers, and the degree of lattice softening with temperature, as observed here, could be used as an indication of matrix-NC bond strength.

**Figure 5:**
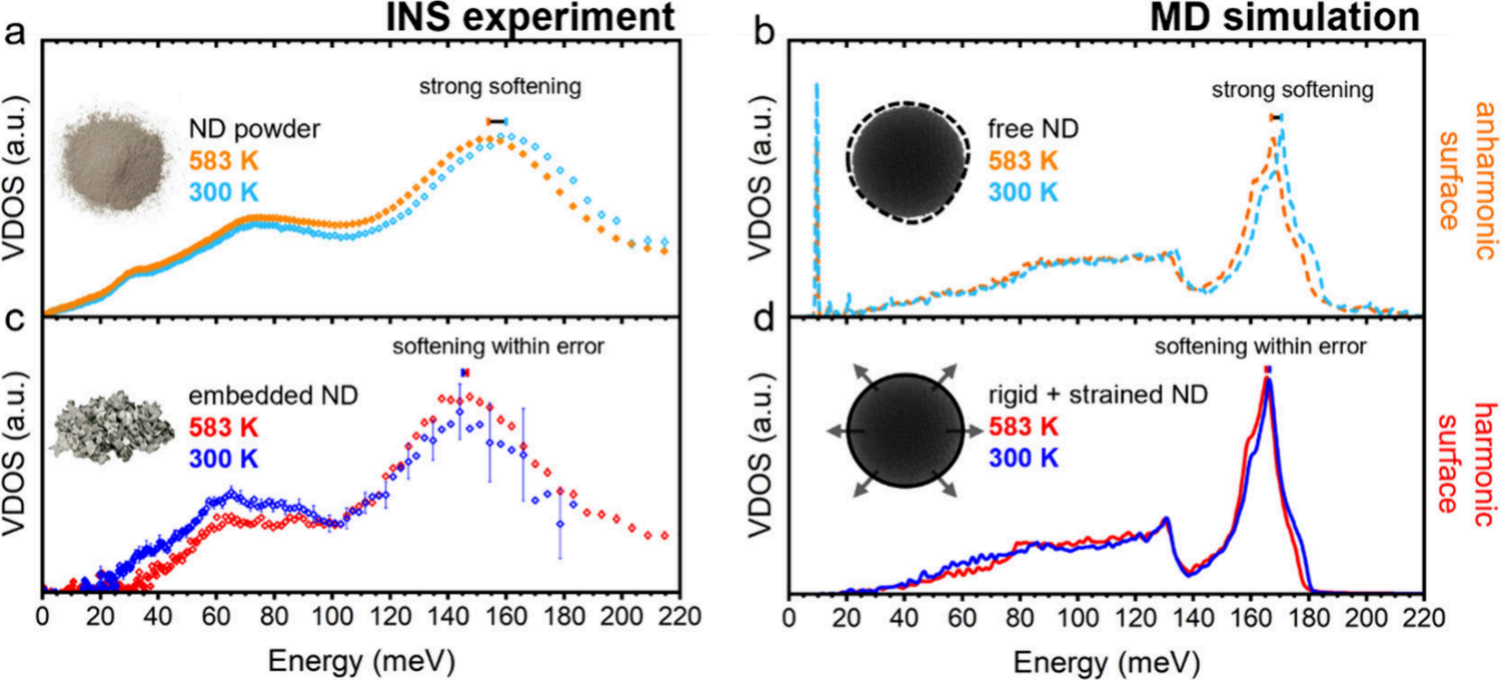
Temperature comparison of the nanodiamond (ND) powder and ND embedded in SnTe vibrational density of states (VDOS) reveals strong phonon anharmonicity related to ND surfaces that are quenched in the matrix by new boundary conditions. (a) VDOS measured at 300 and 583 K for the ND powder and (c) ND embedded in SnTe measured by inelastic neutron scattering (INS) (the large error bars for 300 K are due to the low phonon mode population of high-energy modes). (b) VDOS at 300 and 583 K for a free, isolated ND particle and (d) the same ND particle with rigid boundary conditions and tensile strain (0.01% lattice expansion) (lower panel) simulated by molecular dynamics (MD). Parameters from Gaussian fits of the optical Van Hove singularity in (a) are presented in Table 1.

Our findings provide important insights into thermal transport in nanocomposite materials. When NDs are embedded in SnTe, the quenching of surface-related modes (Rayleigh phonons and Lamb resonances) suggests these modes do not significantly affect interfacial phonon scattering or thermal conductivity. However, the observed core phonon softening from tensile strain likely influences these properties. In ND clusters, some surface modes persist and show anharmonic behavior, potentially affecting thermal transport through clusters and channels, especially in composites where NC percolation occurs. These insights advance our understanding of thermoelectric-nanoparticle composite behavior, with relevance to the promising strategy of using so-called “nanoparticle-in-alloy” materials.^41^

In conclusion, for the first time, the “full” phonon spectrum of a nanocrystal powder with the same nanocrystal embedded in a matrix were measured and compared. With TOF neutron spectroscopy, these phonons can be observed in elementally light NCs, even when the crystals are embedded in a heavy matrix. For NDs embedded in SnTe, the quenching of low-energy surface Rayleigh phonons was observed in the *S*(*Q*,*E*) and VDOS plots. Further analysis of the embedded ND VDOS revealed softer and narrower phonon modes compared to powdered NDs. By performing MD simulations on an isolated ND particle in vacuum and adding simple rigid boundary conditions and tensile stresses, the effects of embedding the NC on its phonon modes were qualitatively reproduced. Subtle differences in the ND VDOS were also demonstrated depending on the average local ND environment, by comparing samples with varying degrees of ND agglomerates. These agglomerates retain in their VDOS a small—but detectable—fraction of surface dynamics. This is evidenced by a slight enhancement of the low-energy VDOS associated with Rayleigh phonons and a slightly soft optical mode VDOS compared to samples with more homogeneous ND dispersion. Finally, at different temperatures, both experiment and simulation revealed highly anharmonic ND surfaces, whose anharmonicity is quenched upon embedding in SnTe.

It is anticipated that many of these features will be universal to NCs embedded in various solid matrices and thus relevant to many phononic, thermoelectric, or similar systems. With the increasing number of uses for NCs being revealed and the importance of their vibrational or thermodynamic properties for many of these applications, it is believed that these results and the development of this experimental framework will have significant benefits for a broad range of research.

## Supplementary Material



## Data Availability

The data supporting the findings of this study are available from the corresponding authors upon request.

## Notes

Additional details of the experimental and simulation methods and additional neutron powder diffraction and simulation data (PDF)

## ∇

School of Chemistry, Monash University, Clayton, VIC 3800, Australia

## §

Institute for Glycomics, Griffith University, Gold Coast, QLD 4215, Australia
