# Supplementary material for: Matrix-Embedding Effects on Nanodiamond Phonons
Source: Nano Lett. 2025 Aug 20;25(35):13087–94. doi: 10.1021/acs.nanolett.5c02276 (PMC12412158; doi:10.1021/acs.nanolett.5c02276)
Supplement: Supplementary file 1 [file nl5c02276_si_001.pdf]

Supplementary Information for

Matrix-Embedding Effects on Nanodiamond Phonons

Caleb Stamper,[1†] David L. Cortie,[1,2] Ablikim Bake,[1‡] Roger A. Lewis,[1] and Dehong Yu[2*]*

[1]School of Physics and Institute for Superconducting and Electronic Materials, University of Wollongong, Wollongong, NSW 2500, Australia.

[2]Australian Nuclear Science and Technology Organisation, Lucas Heights, NSW 2234, Australia.

[*]*Corresponding Authors: dcr@ansto.gov.au and dyu@ansto.gov.au*

Present Addresses: [†]*School of Chemistry, Monash University, Clayton, VIC 3800, Australia.*

[‡]*Institute for Glycomics, Griffith University, Gold Coast, QLD 4215, Australia.*

Methods

Synthesis

SnTe powders (99.99%) were obtained commercially from Xi'an Functional Material Co. and nanodiamond (ND) powders (98.3%) were obtained commercially from US Research Nanomaterials Inc. The 2.4 vol.% and 1.0 vol.% ND doped SnTe and pure SnTe samples were created by hand-mixing weighed powders using a mortar and pestle for ~10 minutes before consolidating. Samples were consolidated via spark plasma sintering (SPS) (Thermal Technology) at 773 K (500 °C) and 50 MPa for 5 minutes (after a fast ramp to 753 K and 40 MPa followed by slower ramp to the final temperature and pressure) using a 14 mm graphite die. The samples were then heat-treated in a tube furnace under a nitrogen gas flow at 773 K for 1 h after a 1 h ramp time and then passively cooled to ensure they had fully undergone any phase transitions. The sample masses used for the INS measurements were 13.4 g, 15.3 g, and 17.6 g for the pure, 2.4% ND, and 1.0% ND SnTe samples, respectively. Thermal diffusivity measurements were taken on another set of samples prepared the same way. These samples were shaped into 6 mm diameter, 1 mm thick discs for measurement.

Structural and Thermal Characterization

A lamella for transmission electron microscopy (TEM) analysis was extracted using a FEI HELIOS G3 microscope. The TEM lamella preparation was carried out using the method reported in the literature.[1] TEM/scanning-TEM (STEM) images were acquired using a JEOL JEM-F200 microscope.

Neutron powder diffraction data were acquired using Echidna,[2, 3] the high-resolution neutron powder diffractometer at the Australian Nuclear Science and Technology Organisation's (ANSTO) Australian Centre for Neutron Scattering (ACNS), with an incident neutron beam wavelength of 1.6221 Å.

Thermal diffusivity measurements were taken using a laser flash analysis (LFA) instrument (Netzsch LFA 467 HyperFlash). The samples were coated with a thin layer of graphite (super-enhanced graphite aerosol) to enhance absorption from the lamp.

Inelastic Neutron Scattering

Inelastic neutron scattering (INS) experiments were conducted on the time-of-flight cold-neutron spectrometer, PELICAN, at the ACNS.[4] The instrument was configured with an incident neutron wavelength of 4.69 Å, corresponding to an incident energy of 3.72 meV and an energy resolution of 0.135 meV at the elastic line. Incident neutrons of half this wavelength (2.345 Å, 14.88 meV) were also used for measurement out to higher values of Q at the cost of resolution and flux. The sintered pellets were broken up and placed in annular aluminium cans with a 1 mm gap to minimize multiple scattering effects. The estimated neutron transmission was 90%. Measurements were taken at 300 K and 583 K using a top-load cryo-furnace. The corresponding empty can was measured in the same conditions as for the samples for background subtraction. In addition, a standard cylinder-shaped vanadium sample was also measured for detector efficiency normalization and energy resolution calibration.

The data reduction, including background subtraction, detector normalization, and determination of the dynamic scattering function $S(Q, E)$, was performed using the Large Array Manipulation Program (LAMP).[5] The phonon density of states was obtained on the neutron energy gain side with normalization to the total area. The DOS was calculated from a minimum energy of 1.2 meV for the nanodiamond sample and 0.4 meV for the microdiamond sample since below these energies the elastic line begins to contribute (with an incident neutron energy of 3.7 meV). The measurements were not corrected for multiple scattering.

Based on the incoherent one-phonon approximation, the PDOS for a Bravais powder sample (isotropic system) can be derived by an INS experiment according to:

$$g_{\text{INS}}(\omega) = C \int \frac{\omega}{Q^2} S(Q, \omega)\left(1 - e^{-\hbar\omega/k_B T}\right) dQ, \quad (1)$$

where C is a factor containing the atomic mass and Debye-Waller factor, $\exp(-2W)$, which is taken as unity for all samples here, k_B is the Boltzmann constant, and $S(Q,\omega)$ is the dynamic scattering function of the sample. The integration over Q covers the experiment-accessible range of momentum transfer.

For a non-Bravais sample, the measured phonon density of states (VDOS) is given by the neutron-weighted phonon density of states:

$$g_{\mathrm{NW}}(\omega) = \sum_i f_i \frac{\sigma_i}{M_i} g_i(\omega)\,, \tag{2}$$

where the sum over i includes all elements in the sample, f_i is the ith atomic concentration, σ_i is the total neutron bound cross-section—as both coherent and incoherent phonon scattering processes are measured experimentally—M_i is the atomic mass, and $g_i(\omega)$ is the partial VDOS of the element i. Since σ_i/M_i is similar for C, Sn, and Te, the DOS roughly represents the true VDOS weighting. We report g as a function of energy (transfer) in the form $E = \hbar\omega_i - \hbar\omega_f$.

Classical Molecular Dynamics

Classical molecular dynamics (MD) simulations were run using the LAMMPS molecular dynamics simulator,[6] following the same general procedure as used previously.[7] Each production simulation was run using a canonical (NVT) ensemble with temperature fixed at 300 K or 583 K. In all ensembles, pressure and temperature were controlled using the Nose-Hoover method.[8, 9]A timestep of 1 fs was used. For production, frames were dumped at a frequency of 1/3 frames/fs (every 3rd frame) to be processed for the vibrational density of states. Atom velocities were adjusted so that the net linear and angular momentum of the particles was zero. The diamond particles were simulated with a 10 ps NVT run (2 ps equilibration, 8 ps for production). We found this to be sufficient time to sample the dynamics of interest in this work after comparing simulations of longer run times. The AIREBO (without Lennard-Jones or torsion contributions) potential was used for all carbon-carbon interactions.[10]

For the free nanodiamond, A spherical diamond particle 5.3 nm in diameter (~13,700 atoms) was simulated in a large vacuum box (~140 nm). To impose Dirichlet boundary conditions on the nanoparticle, the same simulations were run with the surface atoms fixed with force equal to zero. Isotropic tensile strain was imposed on the nanoparticles using the fix deform command in LAMMPS which strains the nanoparticle by changing the simulation volume. Deform parameters were chosen to change the diamond lattice constant by certain fractions for comparison with the experimentally embedded diamonds (see main text).

Visual representations of the MD simulations were created using Visual Molecular Dynamics (VMD).[11]

Phonon Density of States Calculations from MD

The resulting trajectory files from the MD simulations were used to calculate the dynamic quantities presented throughout this work. These calculations were performed using the Molecular Dynamics Analysis of Neutron Scattering Experiments (*MDANSE*) program.[12] The density of states (DOS) is derived by utilizing the velocity autocorrelation function (VACF), *i.e.*, by integrating, over time, all the positional and velocity information of the system. The VACF for an atom in an atomic system is defined as

$$C_{vv;\alpha\alpha}(t) \doteq \frac{1}{3}\langle v_\alpha(t_0)\cdot v_\alpha(t_0+t)\rangle_{t_0}, \tag{3}$$

where $v_\alpha(t)$ is the velocity of atom α as a function of time. For an isotropic system, the DOS (as a function of angular frequency) can be given as

$$g(\omega) = \sum_\alpha b_{\alpha,\mathrm{inc}}^2\, \tilde{C}_{vv;\alpha\alpha}(\omega), \tag{4}$$

where $b_{\alpha,\mathrm{inc}}$ is the incoherent (*i.e.*, single-particle motion) neutron scattering length for atom α, and

$$\tilde{C}_{vv;\alpha\alpha}(\omega) = \frac{1}{2\pi}\int_{-\infty}^{+\infty} e^{-i\omega t} C_{vv;\alpha\alpha}(t)\, dt\,. \tag{5}$$

MDANSE computes the discrete DOS (using the un-normalised VACF so that $g(0)$ approximates the diffusion constant) by:

$$DOS(n \cdot \Delta v) \doteq \sum_{\alpha} \omega_{\alpha}\, \tilde{C}_{vv;\alpha\alpha}(n \cdot \Delta v), n = 0 \ldots N_t - 1, \tag{6}$$

where N_t is the total number of time steps and the frequency step is $\Delta v = 1/(2N_t\Delta t)$. This discrete DOS is then smoothed by applying a Gaussian window (in t), producing the simulated $g(\omega)$.

Ab Initio Molecular Dynamics

Calculations were performed using density functional theory (DFT), deploying the Vienna Ab initio Simulation Package (VASP) based on the projector-augmented-wave (PAW) pseudopotentials method.[13, 14,15] The functional of Perdew, Burke and Ernzerhof (PBE)[16] was used to describe the exchange-correlation energy based on the generalized gradient approximation (GGA). A 2x2x2 diamond supercell was constructed containing 64 carbon atoms. Born Oppenheimer molecular dynamics[17] were performed at 300, 500, 700 and 900 K. The ab initio molecular dynamics simulations used an energy cut-off of 300 eV and a single k-point at the Γ- point. The cell volume was fixed to the experimental lattice constant and the atomic positions were allowed to relax. Thermal equilibration was achieved at each temperature using a velocity-rescaling NVT isostat for 3 picoseconds, before longer 30 picosecond production runs were performed using a canonical ensemble via the algorithm of Nosé. A time step of 1 femtosecond was used for the molecular dynamics' calculations. The energy was converged to within $1\text{x}10^{-6}$ eV for each relaxation step. Comparison calculations were also performed for a 4x2x2 supercell, and with higher planewave energy cutoff (up to 500 eV) to assess the finite size and convergence effects. The settings used were found to provide the best combination of computational accuracy and computational expense.

Supplementary Data

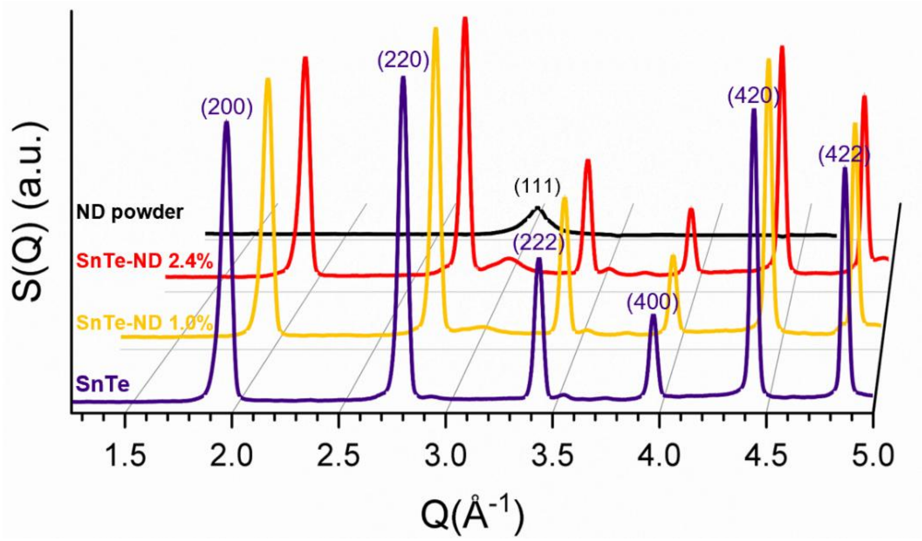

Figure S1. Neutron diffraction patterns for SnTe, nanodiamond (ND) doped SnTe, and pure ND powder. The (111) diamond peak is clearly visible in the 1.0% and 2.4% SnTe-ND composites. Small SnO_2 peaks are present in all SnTe samples. The composite patterns were obtained on the neutron diffractometer Echidna, while the ND powder pattern was taken from the elastic line from data obtained on the time-of-flight spectrometer, Pelican, at ANSTO. All data were collected at 300 K.

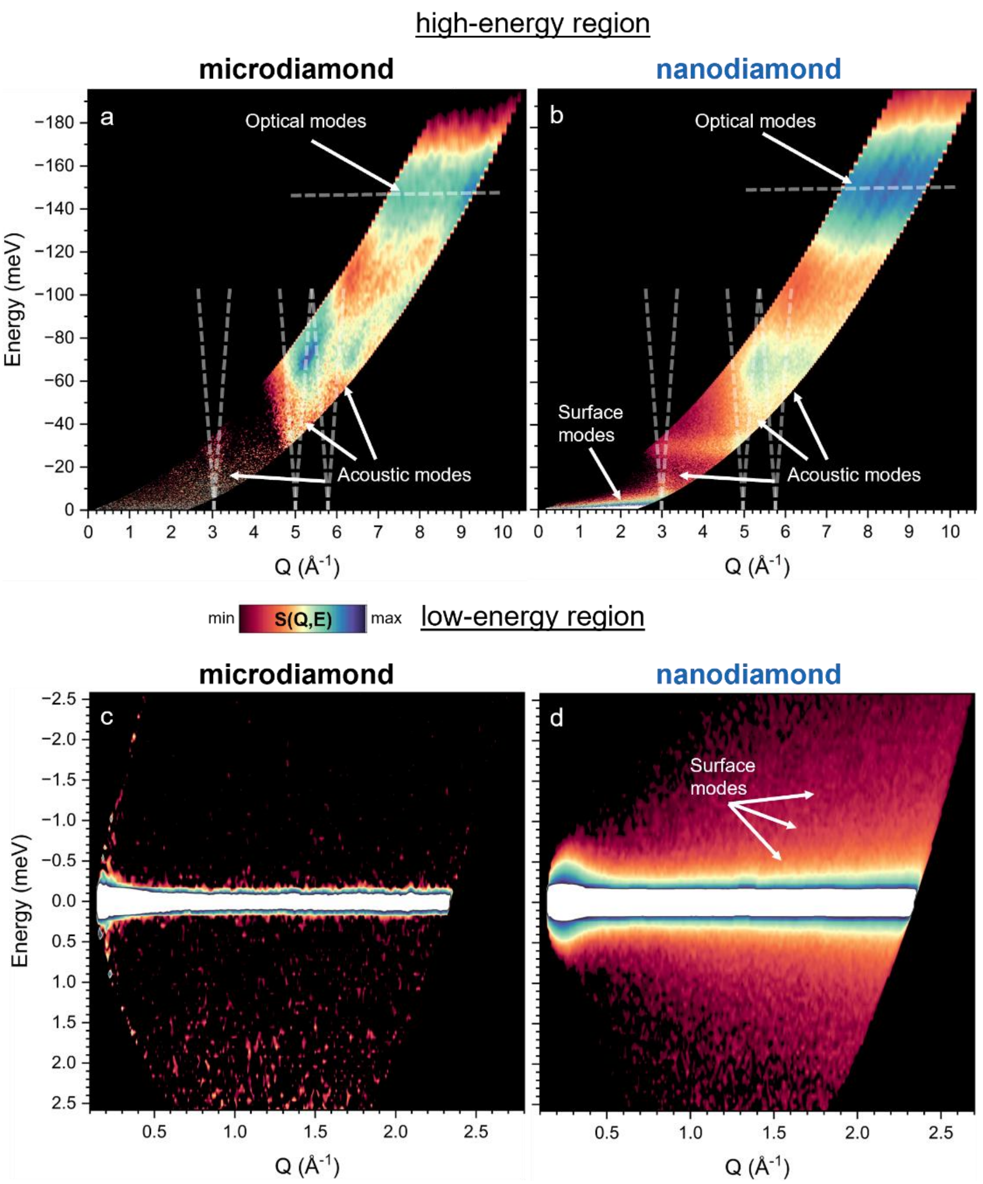

Figure S2. Dynamic structure factor maps from inelastic neutron scattering of microdiamond (pseudo-bulk) (left) (a,c) and the nanodiamond sample (right) (b,d) at 583 K. Dashed lines are given in (a) and (b) as simplified and approximate guides to highlight the origin of the INS signal originating from acoustic and optical phonons. The phonon modes are easier to identify in the bulk sample since they are significantly less broad. Maps (c) and (d) highlight the low-energy signal originating from surface modes. The microdiamond data was collected in the same way as the nanodiamond data and originates from ref. (7).

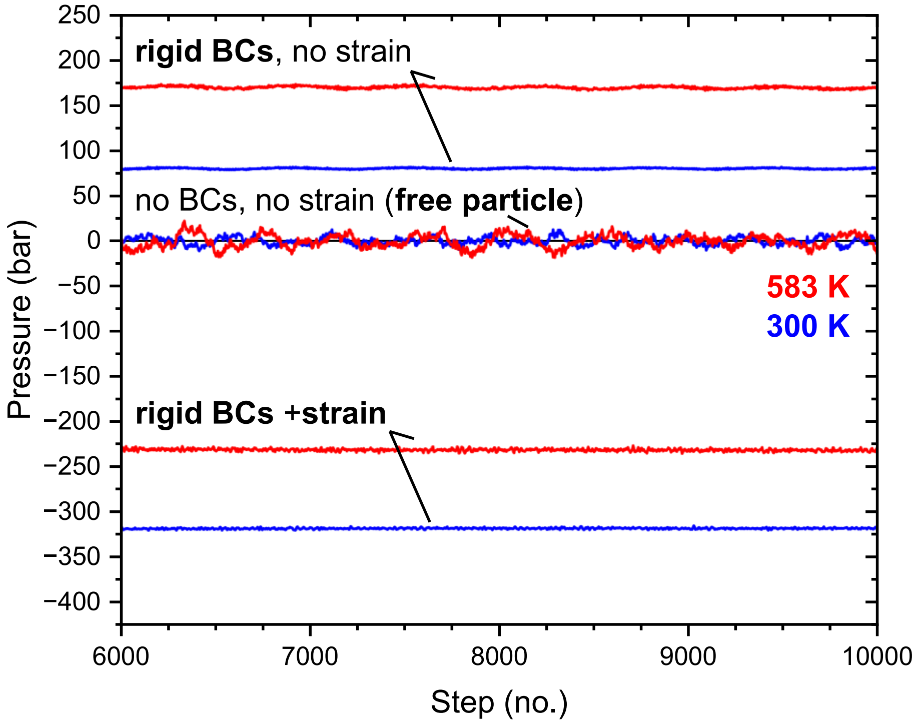

Figure S3. Molecular dynamics simulated nanodiamond particle pressures at 300 K (blue) and 583 K (red) for the free particle, particle under Dirichlet boundary conditions (BCs) (rigid), and the rigid particle with imposed tensile strain (+strain). Pressures are shown over an arbitrary number of stable steps at the end of the simulations.

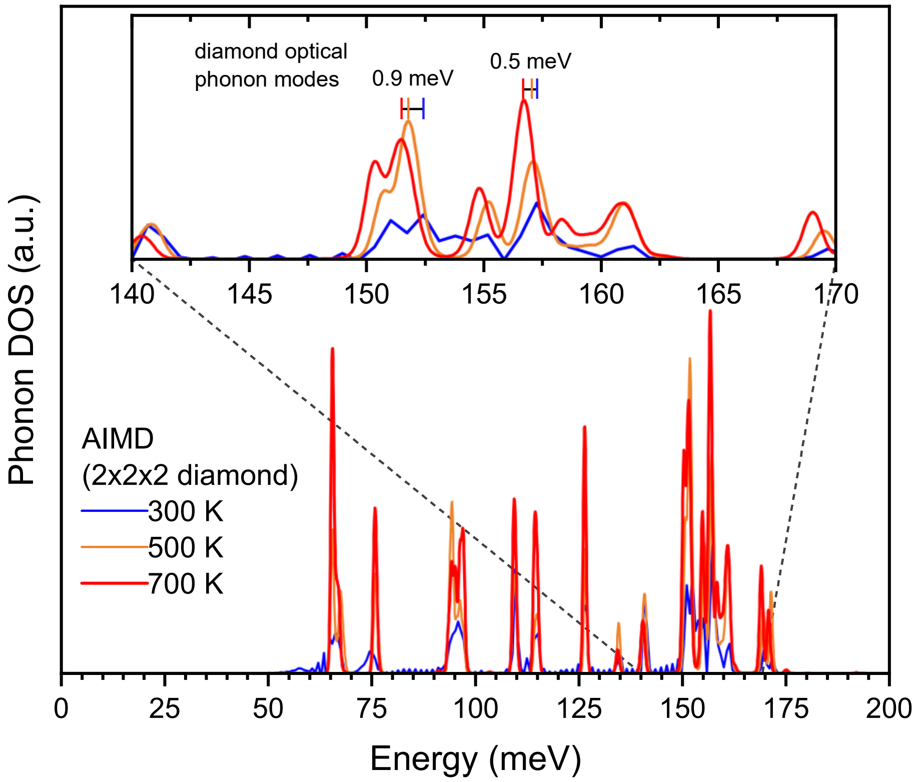

Figure S4. *Ab initio* molecular dynamics (MD) calculated phonon density of states of a 2x2x2 diamond supercell at 300, 500, and 700 K. The inset shows the small degree of optical phonon softening, as is seen experimentally for the embedded nanodiamonds and for the (classical MD) simulated rigid particles, but different from the powdered nanodiamond and free nanodiamond particle simulations where large softening occurs with temperature.

Supplemental References

(1) Bake, A.; Zhao, W.; Mitchell, D.; Wang, X.; Nancarrow, M.; Cortie, D. Lamellae preparation for atomic-resolution STEM imaging from ion-beam-sensitive topological insulator crystals. *Journal of Vacuum Science & Technology A* **2022**, *40* (3). DOI: 10.1116/6.0001771 (acccessed 1/15/2025).
(2) Avdeev, M.; Hester, J. R. ECHIDNA: a decade of high-resolution neutron powder diffraction at OPAL. *Journal of Applied Crystallography* **2018**, *51* (6), 1597-1604.
(3) Liss, K.-D.; Hunter, B.; Hagen, M.; Noakes, T.; Kennedy, S. Echidna—the new high-resolution powder diffractometer being built at OPAL. *Physica B: Condensed Matter* **2006**, *385*, 1010-1012.
(4) Yu, D.; Mole, R.; Noakes, T.; Kennedy, S.; Robinson, R. Pelican—a time of flight cold neutron polarization analysis spectrometer at OPAL. *Journal of the Physical Society of Japan* **2013**, *82* (Suppl. A), SA027.
(5) Richard, D.; Ferrand, M.; Kearley, G. Analysis and visualisation of neutron-scattering data. *Journal of Neutron Research* **1996**, *4* (1-4), 33-39.
(6) Plimpton, S. Fast Parallel Algorithms for Short-Range Molecular Dynamics. *Journal of Computational Physics* **1995**, *117* (1), 1-19. DOI: https://doi.org/10.1006/jcph.1995.1039.
(7) Stamper, C.; Baggioli, M.; Galaviz, P.; Lewis, R. A.; Rule, K. C.; Bake, A.; Portwin, K. A.; Jin, S.; Fan, X.; Yu, D. Thermal Nanoquakes: Terahertz Frequency Surface Rayleigh Waves in Diamond Nanocrystals. *arXiv [cond-mat.mes-hall]* **2024**. DOI: 10.48550/arXiv.2410.07480. (accessed 2024-12-10).
(8) Hoover, W. G. Canonical dynamics: Equilibrium phase-space distributions. *Physical review A* **1985**, *31* (3), 1695.
(9) Nosé, S. A unified formulation of the constant temperature molecular dynamics methods. *The Journal of chemical physics* **1984**, *81* (1), 511-519.
(10) Stuart, S. J.; Tutein, A. B.; Harrison, J. A. A reactive potential for hydrocarbons with intermolecular interactions. *The Journal of Chemical Physics* **2000**, *112* (14), 6472-6486. DOI: 10.1063/1.481208 (acccessed 2/15/2024).
(11) Humphrey, W.; Dalke, A.; Schulten, K. VMD: visual molecular dynamics. *Journal of molecular graphics* **1996**, *14* (1), 33-38.
(12) Goret, G.; Aoun, B.; Pellegrini, E. MDANSE: An Interactive Analysis Environment for Molecular Dynamics Simulations. *Journal of Chemical Information and Modeling* **2017**, *57* (1), 1-5. DOI: 10.1021/acs.jcim.6b00571.
(13) Kresse, G. and J. Hafner, Ab initio molecular-dynamics simulation of the liquid-metal–amorphous-semiconductor transition in germanium. *Physical Review B*, **1994**. 49(20): p. 14251-14269.
(14) Kresse, G. and J. Furthmüller, *E*fficient iterative schemes for ab initio total-energy calculations using a plane-wave basis set. *Physical Review B*, **1996**. 54(16): p. 11169-11186.
(15) Kresse, G. and J. Furthmüller, Efficiency of ab-initio total energy calculations for metals and semiconductors using a plane-wave basis set. *Computational Materials Science*, **1996**. 6(1): p. 15-50.
(16) Perdew, J.P., K. Burke, and M. Ernzerhof, Generalized Gradient Approximation Made Simple. *Physical Review Letters*, **1996**. 77(18): p. 3865-3868.
(17) Kresse, G. and J. Hafner, Ab initio molecular dynamics for open-shell transition metals. *Physical Review B,* **1993**. 48(17): p. 13115-13118.
